# Hand-Assisted Laparoscopic Surgery Is Superior to Open Liver Resection for Colorectal Liver Metastases in the Posterosuperior Segments

**DOI:** 10.3389/fsurg.2021.746427

**Published:** 2021-11-25

**Authors:** Omar Abu-Zaydeh, Muneer Sawaied, Yael Berger, Ahmad Mahamid, Natalia Goldberg, Eran Sadot, Riad Haddad

**Affiliations:** ^1^Department of Surgery, Carmel Medical Center, Haifa, Israel; ^2^Department of Surgery, Rabin Medical Center, Petah Tikva, Israel; ^3^Sackler School of Medicine, Tel Aviv University, Tel Aviv, Israel; ^4^The Ruth and Bruce Rappaport Faculty of Medicine, Technion, Israel Institute of Technology, Haifa, Israel; ^5^Department of Radiology, Carmel Medical Center, Haifa, Israel

**Keywords:** metastatic colon cancer (mCRC), hand-assisted laparoscopic surgery (HALS), laparoscopic liver resection (LLR), posterosuperior segments, open liver resection (OLR)

## Abstract

**Introduction:** Laparoscopic liver resections (LLR) of colorectal metastasis located in posterosuperior segments (1, 4A, 7 and 8) are challenging and highly demanding. The aim of our study is to determine the safety and feasibility of hand-assisted laparoscopic surgery (HALS) in the resections of the posterosuperior lesions and to compare the peri-operative, short-term and long-term outcomes with the open liver resection (OLR) approach.

**Methods and Results:** A retrospective study of patients who underwent either HALS or OLR for metastatic colorectal cancer (mCRC) located in the posterosuperior segments of the liver between 2008 and 2018 in two university affiliated medical centers.

**Results:** A total of 187 patients were identified, of whom 78 underwent HALS and 109 underwent OLR. There was no difference between the HALS and OLR with regard to preoperative factors (age, primary CRC tumor location, number and anatomical distribution of liver metastasis, pre-operative neo-adjuvant treatment, operative time, blood transfusion rate, and resection margins positivity). On the other hand, HALS compared to OLR had a significantly shorter mean hospital stay (4 vs. 6 days; *P* = 0.003), and a lower total complications rate (25 vs. 47% *P* = 0.006). Both groups had no 30-day mortality. Also, patients who underwent HALS vs. OLR had similar liver metastases recurrence (55 vs. 51%. *P* = 0.65) and 5-year survival (47 vs. 45%. *P* = 0.72).

**Conclusions:** HALS for mCRC located in posterosuperior liver segments is safe and feasible and it is a preferable approach due to its lower complication rate and shorter hospital stay while not compromising survival and disease recurrence.

## Introduction

Minimally invasive surgery (MIS) has revolutionized many procedures in different surgical fields over the past three decades. However, the adoption of minimally invasive liver resection has been slow compared with the rate at which MIS approaches have been incorporated into luminal and other solid organ procedures (1–3). This slow adoption has persisted despite expanding indications and increasing experience in high volume centers (4).

Based on international data, primary liver cancer is the most common indication for laparoscopic liver resection (LLR), whereas metastatic disease may predominate as an indication in North America and Europe (5, 6). Of note, LLR is associated with a decrease in perioperative bleeding, faster mobilization, shorter hospital stays, and less morbidity, as well as being cost-effective (7–9). Furthermore, concerns on compromised short- and long-term oncological outcomes in LLR have been refuted by various studies (9–12).

For resection of colorectal liver metastases (CRLM), studies have shown that LLR results in superior perioperative outcomes and similar oncological outcomes (recurrence-free and overall survival) in selected patients compared to open liver resection (OLR) (9–12).

The Louisville consensus conference recommends laparoscopic liver resection for patients with a solitary lesion, 5 cm or less, located in the peripheral liver segments (13). However, in the Southampton guidelines (14) and after accumulated experience in laparoscopic liver surgery for colorectal liver metastases, the warnings and limitations were only for formal laparoscopic hemi-hepatectomy and for lesions in the postero-superior segments. These lesions (in segments 1, 4a, 7, and 8) have been considered especially challenging because of the limited surgical view and restricted handling of laparoscopic instruments (15, 16). However, recent progress in operative techniques, including the introduction of the transthoracic port placement, has reduced the difficulty of LLR for tumors in the posterosuperior liver (17).

Hand-assisted laparoscopic surgery (HALS) is defined as the placement of a hand port during laparoscopy. The main advantages of HALS are the possibility to control intraoperative bleeding via manual compression and the use of tactile sensation, to detect deeper intraparenchymal lesions, and provide better exposure of difficult tumor locations (13, 14, 18).

To date, there is no published comparison of the surgical and long-term results of HALS vs. OLR for CRLM in the postero-superior segments. The aim of this study was therefore to compare the perioperative and long-term outcomes of LLR (HALS) and OLR for CRLM in the postero-superior segments.

## Materials and Methods

All consecutive patients who underwent either HALS or open resections with curative intent for CRLM located in posterior-superior liver segments (segments1, 4a, 7, and 8) between January 2008 and December 2018 were identified from prospectively maintained surgical databases at the Carmel and Rabin medical centers. Patients' data was collected by reviewing these electronic databases of both centers after receiving approval from their institutional review boards. Clinical data including demographic, perioperative and intraoperative variables, in addition to short- and long-term outcomes were collected retrospectively.

The indications for liver resection were determined during a weekly multidisciplinary meeting. Preoperatively the workup included blood tests, tumor markers, imaging modalities [computed tomography (CT), positron emission tomography CT (PET-CT) and magnetic resonance imaging (MRI)], and characterization of the liver metastases (number, location, size, and relation to intrahepatic vascular or biliary structures). All the patients were informed in detail about the procedure, including the risks and benefits, and written consent was obtained before surgery. All the HALS procedures were performed by the same surgical team under the direction of the same attending surgeon (R.H.). Open liver resection were performed by 3 HPB surgeons.

### Surgical Technique

In brief, in the open group, the J-shaped subcostal incision was used, followed by mobilization of the liver and subsequent liver resection as standard.

In the HALS group, the procedure was performed as described earlier by Sadot et al. (19). Three trocars (two 12-mm and one 5-mm) were inserted in the upper midline abdomen, and a hand-assisted device (GelPort, Applied Medical, CA, USA) was placed in the right abdominal horizontal incision (7–8 cm). The pneumoperitoneum was generated with CO_2_ at a pressure of 12–15 mmHg, and visual exploration of the abdominal cavity was conducted with a 30° laparoscope. Intra-abdominal sonography of the liver was performed in order to plan the liver resection, followed by mobilization of the liver according to the location of the lesions. The liver resections were carried out using a 5-mm bipolar sealing device (LigaSure Dolphin tip; Valleylab, Boulder, CO, USA), Endo GIA™ staplers (vascular cartridge, Endo GIA™, Covidien, Norwalk, CT, USA), and the Cavitron Ultrasonic Surgical Aspirator (CUSA; Valleylab, Boulder, CO, USA). The specimen was then extracted through the hand-assisted device without a bag. The surgical field was irrigated and checked for bleeding or bile. Before completion of the operation, central venous and blood pressure were restored to normal parameters to confirm hemostasis. An abdominal Jackson-Pratt (JP) drain was usually placed through a 5-mm port site. The wounds were then closed in layers after deflation of the pneumoperitoneum. All specimens were sent fresh for pathologic examination to measure the surgical margins.

Blood loss was estimated using the volume of blood aspirated from the abdominal cavity during the procedure. Operative time was defined as the time elapsed from the skin incision until closure. Postoperative hospital stay was defined as the number of hospitalized days from the first day after operation until the day of discharge. From the pathological report, we collected the data on the number of metastases, the largest tumor's diameter, and the narrowest margin distance for each patient. R0 was defined as no cancer cells seen microscopically at the resection margin. Complications were classified according to the Clavien-Dindo grading system. Clinical risk score (CRS) was stratified into two groups: the first group was composed of patients with a low CRS (0–2 points), and the second group was composed of patients with a high CRS (3–5 points).

During the follow-up period, the patients were followed by our multidisciplinary team during the first month, every 4 months in the first 2 years, and then twice a year. Follow-ups included clinical examinations, blood work-up including carcinoembryonic antigen (CEA), and spiral CT of the chest-abdomen or PET-CT.

### Statistical Analysis

Data were analyzed using the Statistical Product and Service Solutions 25.0 package for Macintosh (SPSS Inc., Chicago, IL, USA). Results were presented as means and standard deviations. The overall survival and disease-free survival were determined using the Kaplan Meier method. Comparisons were made using the c2 test or one-way ANOVA for categorical or continuous variables, respectively, and a *p*-value < 0.05 was considered as statistically significant.

## Results

### Study Population

During the study period, 187 patients with mCRC located in the posterior-superior liver segments were identified; 78 of them underwent HALS and the remaining 109 patients underwent OLR. The characteristics of the patients including their demographic and pre-operative clinical data are shown in Table 1. Both patient groups had a comparable mean age (OLR 66 ± 11 years vs. HALS 64 ± 12 years; *p* = 0.64) and a comparable division of the sexes (OLR 58% males vs. HALS 47%; *p* = 0.26).

**Table 1:** Baseline demographical, liver and colorectal characteristics.

	**Laparoscopic (*n* = 78)**	**Open (*n* = 109)**	***p*-value**
Age (years) mean	64 ± 12	66± 11	0.64
Gender			0.26
Male	37 (47.4%)	57 (58%)	
Female	41 (52.4%)	52 (42%)	
Primary tumor location			0.81
Right colon	25 (32%)	34 (31%)	
Left colon	26 (33%)	38 (35%)	
Rectum	26 (33%)	34 (31%)	
**Liver tumor**
Median size of largest metastases (mm)	20 (13:32)	24.5 (15;35)	0.19
Number of metastases			0.9
1	40 (51%)	54 (52%)	
2	15 (19%)	26 (25%)	
>3	23 (30%)	24 (23%)	
Number (median)	1 (1; 2)	1 (1; 2)	0.93
Synchronous	40 (51%)	49 (45%)	0.37
Metachronous	38 (49%)		
Clinical risk factor			0.78
Low	67 (86%)	92 (84%)	
High	11 (14%)	17 (16%)	
Neoadjuvant therapy	56 (72%)	69 (63%)	0.24

The anatomical distribution of the primary colorectal tumors did not show statistically significant differences (*p* = 0.95) between the two groups; most of the tumors in both groups were located in the right-side colon (43%). Another one-fifth of the patients presented with rectal cancer (laparoscopic group 19% vs. open surgery group 21%).

When considering liver metastases, the patient groups had comparable pathology. Specifically, there were no statistically significant differences in the median size of the largest liver metastases from the pathological specimens (OLR 24.5 mm vs. HALS 20 mm; *p* = 0.19); both patient groups had a median number of 1 metastasis (*p* = 0.93); and both groups 50% of the patients had more than one metastasis. Moreover, there was no significant group difference in the proportion of patients with a high CRS score (OLR 16% vs. HALS 18%; *p* = 0.78). Lastly, a comparable proportion of patients in each group received neoadjuvant chemotherapy [OLR 69 (63%) patients vs. HALS 55 (72%) patients; *p* = 0.24].

### Clinicopathologic Characteristics of the Patients' Liver Lesions

The patients' laparoscopy or open surgery liver resection procedures of mCRC liver metastases are detailed in Table 2. Both patient groups underwent non-anatomical parenchymal sparing liver resections. There was no difference in the anatomical distribution of the liver metastases between the OLR and HALS groups. However, 33 (42%) patients in the HALS group and 39 (36%) in the OLR group had multiple liver resections in the postero-superior segments.

**Table 2:** Surgical and histological results.

	**Laparoscopic (*n* =78)**	**Open (*n* = 109)**	***p*-value**
Type of liver resection			0.17
Segment 1	2 (2.5%)	2 (2%)	
Segment 4A	7 (9%)	19 (17%)	
Segment 7	16 (20.5%)	27 (25%)	
Segment 8	14 (18%)	11 (10%)	
B/W Segment 7–8	6 (8%)	11 (10%)	
Mix 7, 8, 4A	33 (42%)	39 (36%)	
Conversion	2 (3%)		
Simultaneous colon resection	9 (12%)	17 (16%)	0.52
Operative Time	251 ± 148	266 ± 132	0.49
Blood Transfusion	18 (23%)	35 (32%)	0.11
30-day mortality	0 (0%)	0 (0%)	
Complications	20 (25%)	51 (47%)	0.003
Clavien Dindo I-II	13 (16%)	24 (22%)	
Clavien Dindo III-IV	7 (9%)	27 (25%)	0.006
Hospital stay (days)	4 (4; 6)	6 (4; 8)	0.003
Liver metastasis surgical margins
R0	69 (88.5%)	88 (81%)	0.24
Adjuvant chemotherapy	58 (74%)	69 (63%)	0.34

### Perioperative Outcome

Perioperative outcomes are shown in Table 2. The total operation time was comparable between the two patient groups; 266 ± 132 min in the OLR vs. 251 ± 148 min in the HALS group (*p* = 0.49). The transfusion rate was also comparable; OLR 35 (32%) patients vs. HALS 18 (23%) patients; *p* = 0.11. In the HALS group, conversion was required for two patients (3%) due to bleeding during liver transection.

The length of hospital stay was significantly shorter in the HALS group (median 4 days) than in the OLR group (median 6 days); *p* = 0.003. No death within 90 days after hepatectomy was observed in either group.

The overall postoperative morbidity rate was significantly lower in the HALS group compared to the OLR group (25 vs. 47%; *p* = 0.003). The OLR group also showed a significantly higher rate of major complications “Clavien Dindo III-IV” (25 vs. 9%; *p* = 0.006). Nine patients (4.8%) had bile leek in both groups, the rate was 4.6% in OLR vs. 5.1% in LLR (*p* = 0.56). No patients need re-laparotomy due to bleeding. Finally, an R0 resection was achieved in 69 patients (89%) in the HALS group compared to 88 patients (81%) in the OLR group (*p* = 0.24), with a mean histological tumor-free margin of 7.2 ± 5.8 mm vs. 6.4 ± 6 mm in the HALS and OLR groups, respectively, (*p* = 0.44).

### Overall and Disease-Free Survival

**Figure 1:**
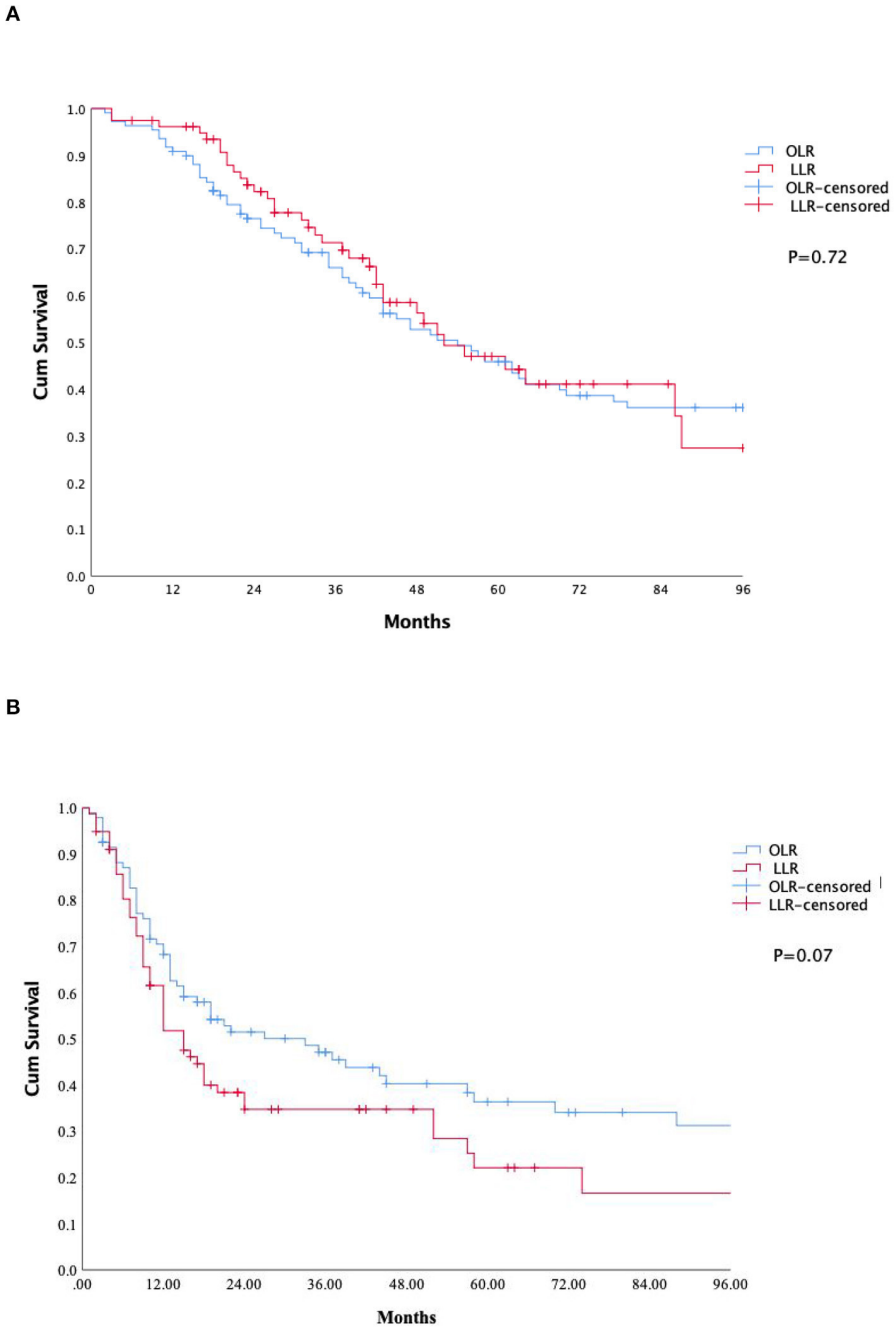
Kaplan-Mayer curve survival. **(A)**: Overall survival. **(B)**: Recurrence free survival. OLR, Open liver resection; LLR, laparoscopic liver resection.

Survival analyses are shown in Table 3. The median follow-up period was similar between the OLR and HALS groups (41 vs. 40 months, *p* = 0.32). The 5-year disease free survival, overall survival rate was similar in both the OLR and HALS groups (36 vs. 22%, *p* = 0.07 and 0.45 vs. 47%, *p* = 0.72 respectively) (see Figure 1).

**Table 3:** Short- and long-term outcomes.

	**Laparoscopic (*n* = 78)**	**Open (*n* = 109)**	***p*-value**
Median follow-up (months)	40 (23; 56)	41 (19; 92)	0.32
Overall survival
Median (months)	52	54	0.72
12 (months)	96%	91%	
24 (months)	84%	76%	
36 (months)	71%	66%	
60 (months)	47%	45%	
Liver recurrence	43 (55%)	56 (51%)	0.65
Status			0.11
Alive	42 (54%)	63 (58%)	
Dead	36 (46%)	46 (42%)	

Liver disease recurrence occurred in 43 (55%) patients in the HALS group and 56 (51%) patients in the OLR group (*p* = 0.65).

## Discussion

The findings of this study demonstrate that the laparoscopic approach is safe, feasible and preferable for resection of mCRC located in the postero-superior segments of the liver. This was shown by a significantly shorter hospital stay and lower complication rate in the laparoscopic group compared to the open surgery group. Liver metastases recurrence and 5-year survival were similar in both patient groups.

Surgical resection remains the only effective treatment for mCRC to the liver. In addition, in the last decade there has been a shift toward parenchymal-sparing surgery which has become the gold standard for the treatment of colorectal liver metastases. The advantages of this approach are in preserving healthy remnant liver parenchyma and maintaining the liver vasculature anatomy. Accordingly, multiple liver resections and repeated hepatectomies can be carried out if needed. Parenchymal-sparing surgery has subsequently decreased the rate of morbidity and mortality and repeated hepatectomies when needed are associated with improved long-term survival.

The three consensus guidelines [Louisville (13), Morioka (7) and Southampton (14)] on laparoscopic liver resections estimate that pure laparoscopic liver resection, HALS, and the hybrid technique appear to be equivalent and are just a matter of surgeon preference. It has also been suggested that the HALS technique may be used to manage intraoperative difficulties and act as a bridge from learning open procedures to laparoscopy surgeries.

Historically, LLR was advocated for liver lesions located in the anterolateral segments, while the open approach was preferred for posterosuperior lesions. With increasing experience, LLR of more challenging lesions in the postero-superior segments has become possible (20). However, in the Southampton 2018 guidelines there were warnings and limitations for formal laparoscopic hemi-hepatectomy and for lesions in the postero-superior segments (14). These warnings were because of concerns regarding the limited visualization of the posterior surface of the liver, the risks of bleeding since the postero-superior segments are adjacent to the major liver vasculature, and the difficulty to control bleeding.

In conventional open liver surgery for mCRC, large incisions are usually required. Whereas, the benefits of liver minimal-invasive surgery are mainly related to smaller incisions with less operative trauma, reduced levels of postoperative pain, and faster gain of functional recovery (20). These benefits are the main reason for shortening the time to resume the adjuvant chemotherapy.

In our experience, HALS has been used during the learning curve as a bridge to master the pure LLR. However, it has now been abandoned for minor hepatic resections while still being valuable for major resections and tumors located in the postero-superior segments. In addition, only after we gained experience in HALS-liver resections of “laparoscopic segments” and were comfortable with the technique, did we then begin to perform HALS-resections of mCRC in the postero-superior segments. The main advantages of HALS over LLS, especially in the parenchymal preserving resection of lesions in the poster-superior segments, are: (i) the faster mobilization of the liver; (ii) the better exposure of difficult metastases locations; (iii) the use of tactile sensation for the detection of subcapsular lesions or superficial small diminished lesions after neoadjuvant chemotherapy; (iv) the better identification of safe resection margins; and (v) the possibility to achieve faster hemostasis of intra-operative bleeding through manual compression. It is worth noting that in minimal invasive liver resections, a 6–8 cm incision is needed to retrieve the resected liver. In pure laparoscopy, a Pfannenstiel incision is made; whereas in HALS, a transverse incision in the upper right abdomen is required but the operative trauma remains significantly reduced compering to OLR. Indeed, Wabitsch et al. (21) reported no significant differences in HALS vs. LLR at operation time, postoperative complications, microscopic surgical margin involvement (R1) rate and general hospitalization time, but they did recommend the implementation of HALS before LLR in the postero-superior segments.

This study's findings showed that despite the technical difficulties of HALS-LLR for patients with postero-superior lesions, there were no significant differences compared to patients undergoing OLR in short-term outcomes, including global operative time and blood transfusion rates. However, there was a significant advantage of the HALS in terms of fewer total and major complications and shorter hospital stays. These results can be partly explained by the fact that LLR for posterosuperior segments were performed in our unit at a later stage of the implantation of the minimally invasive surgery (MIS) liver program. Notably, our findings are aligned with the results of a subgroup analysis of tumors in the postero-superior segments from the first randomized controlled trial, the OSLO-COMET, that compared the outcomes of laparoscopic and open parenchymal-sparing liver resections for mCRC (22). Our findings are also similar to the results of a meta-analysis of 11 studies to compare the outcomes of OLR vs. LLR for mCRC and primary liver tumors in the postero-superior segments (21). This meta-analysis found no group differences in the need for blood transfusions and mortality, but the LLR group had a lower risk of total and major complications and a shorter hospital stay compared to the OLR group. Several additional studies have also reported the advantage of LLR compared to open surgery in terms of lower rates of total and major complications (23–33).

One of the major concerns in MIS liver resection is oncological outcome, especially the achievement of adequate free surgical margins. We found that using the HALS combined with meticulous laparoscopic intraoperative ultrasonography did not compromise the early oncological outcomes, and the rate of R0 margin was not statistically different between the OLR and HALS groups. This is in line with the results reported in the OSLO-COMET study (22) and in a meta-analysis conducted by Hajibandeh et al. (34).

Notably, the safety and feasibility of the HALS did not compromise long-term outcomes. Furthermore, there were no significant differences between the OLR and LLR patient groups in their 3 and 5-year overall survival and in the rate of liver recurrence. This is again in line with the results reported in the OSLO-COMET study (22) and in the Hajibandeh et al. (34) meta-analysis.

There are a few limitations to the study. These include the retrospective data analysis and selection bias. In addition, all the LLR surgeries were performed by one hepatobiliary surgeon, while all the OLR operations were performed by three hepatobiliary surgeons. This creates some heterogenicity between the techniques employed for the parenchymal transections including the use of different devices.

## Conclusions

HALS for CRLM in the postero-superior segments was associated with fewer total and major complications, a shorter hospital stay and similar oncological outcomes compared to open liver resection.

## Data Availability Statement

The raw data supporting the conclusions of this article will be made available by the authors, without undue reservation.

## Ethics Statement

The studies involving human participants were reviewed and approved by Carmel Medical Canter and Rabin Medical Center. Written informed consent for participation was not required for this study in accordance with the national legislation and the institutional requirements.

## Author Contributions

OA-Z, MS, NG, and RH contributed to conception and design of the study. OA-Z, MS, and YB organized the database. AM and NG performed the statistical analysis. OA-Z wrote the first draft of the manuscript. All authors contributed to the article and approved the submitted version.

## Conflict of Interest

The authors declare that the research was conducted in the absence of any commercial or financial relationships that could be construed as a potential conflict of interest.

## Publisher's Note

All claims expressed in this article are solely those of the authors and do not necessarily represent those of their affiliated organizations, or those of the publisher, the editors and the reviewers. Any product that may be evaluated in this article, or claim that may be made by its manufacturer, is not guaranteed or endorsed by the publisher.
